# Guillain–Barré syndrome risk among individuals infected with Zika virus: a multi-country assessment

**DOI:** 10.1186/s12916-018-1052-4

**Published:** 2018-05-15

**Authors:** Luis Mier-y-Teran-Romero, Mark J. Delorey, James J. Sejvar, Michael A. Johansson

**Affiliations:** 1 Division of Vector-Borne Diseases, Centers for Disease Control and Prevention, San Juan, Puerto Rico; 2 Division of Vector-Borne Diseases, Centers for Disease Control and Prevention, Fort Collins, CO USA; 3 Division of High-Consequence Pathogens and Pathology, National Center for Emerging and Zoonotic Infectious Diseases, Centers for Disease Control and Prevention, Fort Collins, CO USA; 4 T.H. Chan School of Public Health, Harvard University, Cambridge, MA USA

**Keywords:** Guillain–Barré syndrome, Zika virus, neurological disorder, vector-borne diseases

## Abstract

**Background:**

Countries with ongoing outbreaks of Zika virus have observed a notable rise in reported cases of Guillain–Barré syndrome (GBS), with mounting evidence of a causal link between Zika virus infection and the neurological syndrome. However, the risk of GBS following a Zika virus infection is not well characterized. In this work, we used data from 11 locations with publicly available data to estimate the risk of GBS following an infection with Zika virus, as well as the location-specific incidence of infection and the number of suspect GBS cases reported per infection.

**Methods:**

We built a mathematical inference framework utilizing data from 11 locations that had reported suspect Zika and GBS cases, two with completed outbreaks prior to 2015 (French Polynesia and Yap) and nine others in the Americas covering partial outbreaks and where transmission was ongoing as of early 2017.

**Results:**

We estimated that 2.0 (95% credible interval 0.5–4.5) reported GBS cases may occur per 10,000 Zika virus infections. The frequency of reported suspect Zika cases varied substantially and was highly uncertain, with a mean of 0.11 (95% credible interval 0.01–0.24) suspect cases reported per infection.

**Conclusions:**

These estimates can help efforts to prepare for the GBS cases that may occur during Zika epidemics and highlight the need to better understand the relationship between infection and the reported incidence of clinical disease.

**Electronic supplementary material:**

The online version of this article (10.1186/s12916-018-1052-4) contains supplementary material, which is available to authorized users.

## Background

The ongoing epidemic of Zika in the Americas has highlighted two serious potential consequences of Zika virus (ZIKV) infection, namely congenital Zika syndrome and Guillain–Barré syndrome (GBS) [1, 2]. Here, we focus on GBS, a condition of acute muscle and limb weakness due to immune-mediated damage to the peripheral nerves. Evidence suggests that GBS arises from autoimmune processes that may be triggered by various antecedent antigenic stimuli, including bacterial or viral infections [3, 4]. Though many cases, particularly among younger patients, undergo full recovery, severe cases require intensive treatment, including respiratory assistance and administration of definitive therapy comprising intravenous immunoglobulins or large-volume plasma exchange [5–7].

A meta-analysis of studies in North America and Europe found (1) that average GBS incidences range from 0.81 to 1.89 cases per 100,000 population per year; (2) that the average risk grows by 20% for every 10-year increase in age; and (3) that GBS is more common in males than females, with a relative risk of 1.78 (95% confidence interval (CI) 1.36–2.33) [4]. A potential connection between ZIKV infection and GBS was first noted during an outbreak of Zika in French Polynesia [8]. It was later shown that all 42 reported GBS cases in French Polynesia had evidence of previous ZIKV infection, as compared with approximately half of non-GBS controls [9]. A retrospective serosurvey in French Polynesia estimated that approximately 49% of the general population and 66% of schoolchildren had been infected by ZIKV, including persons with symptomatic and asymptomatic infections [10]. Thus, with 42 reported GBS cases, it was estimated that the risk of GBS due to ZIKV infection was approximately 2.4 cases per 10,000 ZIKV infections [9].

The current Zika outbreak in the Americas has given rise to confirmed local ZIKV transmission in 47 countries or territories where transmission had never been previously reported [1]. As of March 10, 2017, 22 countries or territories in the Americas (Brazil, Bolivia, Colombia, Costa Rica, Curaçao, Dominican Republic, El Salvador, French Guiana, Grenada, Guadeloupe, Guatemala, Haiti, Honduras, Jamaica, Martinique, Mexico, Panama, Puerto Rico, Saint Martin, Suriname, Trinidad and Tobago, and Venezuela) had reported notable increases in GBS incidence or GBS potentially linked to ZIKV infections. Empirically, many of these increases were spatio-temporally correlated with reports of Zika cases and numerous reported GBS cases had confirmed or suspected links to ZIKV infection [11, 12].

While a causal link between ZIKV infection and GBS has not been established, there are now multiple observations of (1) increased GBS incidence coinciding with ZIKV outbreaks, (2) a decline in GBS incidence once a ZIKV outbreak has concluded, and (3) ZIKV infection documented in some of the reported GBS cases.

Estimates of incidence of GBS in the setting of ZIKV infection are critical for public health decision-making and to ensure the allocation of appropriate medical interventions, such as ventilators and intensive care unit hospital beds, and the provision of definitive interventions, including intravenous immunoglobulins and large-volume plasma exchange. Here, we used the limited data from nine locations in the Americas that reported an increase in reported GBS cases (Bahia state in Brazil, Colombia, Dominican Republic, El Salvador, Honduras, Puerto Rico, Salvador city in Bahia, Brazil, Suriname, and Venezuela) and data from previous outbreaks (Yap and French Polynesia) to estimate the total incidence of ZIKV infection at each location and, from this, the elevated risk of GBS that may be associated with ZIKV infection.

## Methods

This study used data from 11 locations with confirmed Zika outbreaks and reports of potentially associated GBS cases, specifically Yap [13], French Polynesia [9], Bahia state in Brazil [11], Colombia [14], Dominican Republic [15], El Salvador [11], Honduras [11], Puerto Rico [16], Salvador city in Brazil [17], Suriname [11], and Venezuela [11]. The data for Yap and French Polynesia comprises a full outbreak, whereas the data for the other nine locations do not. Herein, ‘ZIKV infections’ refers to the total number of individuals infected with ZIKV, which is not actually observed and is independent of reporting. Unless otherwise specified, ‘Zika cases’ or ‘GBS cases’ refer to reported suspect cases, which are observed but may be misclassified. For each location, we used the most recent, publicly available reported suspect GBS case data for time periods during the outbreaks (Table 1). Notably, all GBS cases reported in Colombia and Puerto Rico displayed symptoms compatible with ZIKV infections. To capture the incidence of reported Zika cases, we used data on reported suspect Zika or arboviral disease cases (in the case of Puerto Rico) for the same time period for each location. Despite limited specificity and varying surveillance systems [18], suspect cases were used for consistency and variation in surveillance was considered in the model framework.

**Table 1:** Population and epidemiological parameters for the Zika outbreaks

	Population (thousands)	EW of dataset	Time period (weeks)	Reported suspect GBS cases	Reported suspect Zika cases	Source
Bahia, Brazil	15,203	EW1–2015 to EW52–2015	52	155	30,266	[11]
Colombia	48,230	EW42–2015 to EW52–2016	62	677	105,027	[14]
Dominican Republic	10,400	EW3–2016 to EW52–2016	50	285	5241	[15]
El Salvador	6426	EW37–2015 to EW13–2016	28	184	11,054	[11]
French Polynesia	280	EW41–2013 to EW15–2014	27	42	31,448	[23]
Honduras	8423	EW1–2016 to EW13–2016	13	71	17,485	[11]
Puerto Rico	3600	EW1–2016 to EW7–2017	59	68	73,034	[16]
Salvador, Brazil	2700	EW7–2015 to EW52–2015	46	49	16,966	[17]
Suriname	548	EW38–2015 to EW13–2016	28	15	3097	[11]
Venezuela	31,292	EW49–2015 to EW13–2016	17	684	32,801	[11]
Yap, Micronesia	7.391	EW16–2007 to EW28–2007	13	0	180	[13]

Population size for Yap from [13]; for Salvador, Brazil from [24]; all others from [11, 25]

*EW* epidemiological weeks, *GBS* Guillain–Barré syndrome

We formulated a Bayesian inference framework that considers the probabilities of each outcome of interest, as follows:

1. A location-specific probability of ZIKV infection, *p*_*Z*_, indicates the proportion of the total population infected over a specified time period.
2. Each person infected by ZIKV has a possibility of experiencing symptoms and presenting as a clinical case, which could be reported to a surveillance system as a suspect Zika case (some systems report only suspect arboviral disease cases; for the purpose of this manuscript, we consider these as suspect Zika cases). We characterized the relationship between suspect clinical cases and ZIKV infection as *p*_*S*_, indicating the number of suspect Zika cases per ZIKV infection that are ultimately reported. To capture the high variability in surveillance systems and healthcare-seeking behavior, we allowed *p*_*S*_ to be location specific, but constrained within bounds that are common across all locations. These location-independent bounds provide a generalized estimate of *p*_*S*_ and also couple our estimates across locations.
3. Each person infected by ZIKV also has a possibility of becoming a reported GBS case with a probability *p*_*GZ*_. Because there may be location-specific differences in reporting, we assumed that *p*_*GZ*_ had some location-dependence constrained within bounds that are common across all locations. These bounds allow for a generalized risk estimate of *p*_*GZ*_ based on data from all locations and considering location-specific variability in risk and surveillance.
4. GBS can also arise from other causes independently of ZIKV infection, or from both simultaneously. We assumed that the baseline rate of reported GBS, *p*_*GBS baseline*_, would follow estimated population-level mean risk of 1.1 cases per 100,000 people per year with a 95% confidence interval (CI) of 0.8–1.9 cases per 100,000 people per year [4]. Because the time periods of the data varied across locations, we converted the baseline risk estimate to a weekly rate and applied it to the time period covered for each location.
5. For Colombia and Puerto Rico, GBS was only reported if preceded by symptoms compatible with ZIKV infection or laboratory confirmation of ZIKV infection. We thus assumed that the total number of GBS cases reported represented only people infected with ZIKV. For other locations where this distinction was not clear, we assumed that the total number of GBS cases observed was the sum of the cases arising from ZIKV infection (3) and the baseline risk (4).

We developed a Bayesian inference framework relating these probabilities to the observed data via a binomial sampling process. We then estimated *p*_*Z*_, *p*_*S*_, and *p*_*GZ*_ using Markov Chain Monte Carlo sampling and implemented our model in JAGS 4.1.0 through the package ‘rjags’ in R. Full details of the model and model convergence diagnostics are available in the supplementary material (Additional file 1).

## Results

**Fig. 1:**
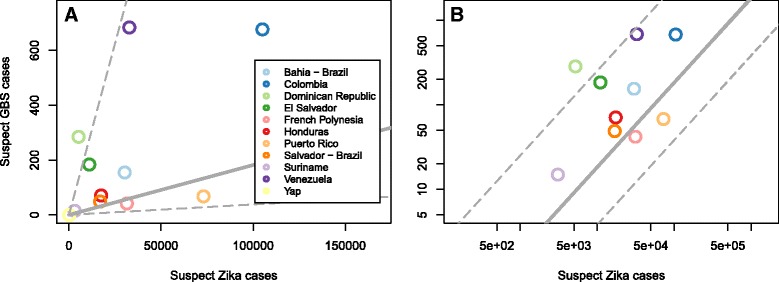
Suspect GBS and Zika case data at the 11 locations that we consider on a linear scale (**a**) and a log-log scale (**b**); note that Yap is missing from panel ‘b’ because no GBS cases were detected there. Using the raw GBS and case data (**a**) there is a positive though not statistically significant correlation of 0.54 (Pearson correlation, 95% confidence interval −0.08 to 0.86). The model, however, considered the uncertainty and variability in these observations and showed a significant relationship indicated by grey lines for the estimated median number of reported suspect GBS cases for a given number of reported suspect Zika cases in an unspecified location (solid) and the 95% credible interval of that estimate (dashed)

In the 11 locations and corresponding time periods considered, a total of 2230 GBS cases were reported (Table 1). The locations with more reports of suspect Zika cases generally reported more suspect GBS cases, though there was also substantial variability in this relationship (Fig. 1). The outbreak in French Polynesia from October 2013 to April 2014 had overall ZIKV infection incidence estimates from two serosurveys [10]. The first was carried out while the outbreak was waning, wherein 196 participants aged 7–86 from all five archipelagos were enrolled between February and March of 2014, with a reported 49% (95% CI 42–57%) seroconversion. The second, in May and June of 2014, when the outbreak had subsided, enrolled 476 schoolchildren in Tahiti, the largest island, and resulted in a seroconversion estimate of 66% (95% CI 60–71%). Because of the spatially and temporally limited samples and variability between them, we conservatively considered the whole range from the two studies (42–71%) as the most likely range of overall incidence for all of French Polynesia. The outbreak in Yap spanned from April through July of 2007, and a post-outbreak serosurvey enrolled all household members of at least 3 years of age in 200 households. This study estimated that 73% (95% CI 68–77%) of the population had been infected [13]; we used this 95% CI as the plausible range for the infection rate in Yap. For the other locations, for which the data does not cover a complete outbreak and serosurveys were not available, we made a prior assumption that the proportion of the population infected was unknown and therefore between 0 and 100%.

**Fig. 2:**
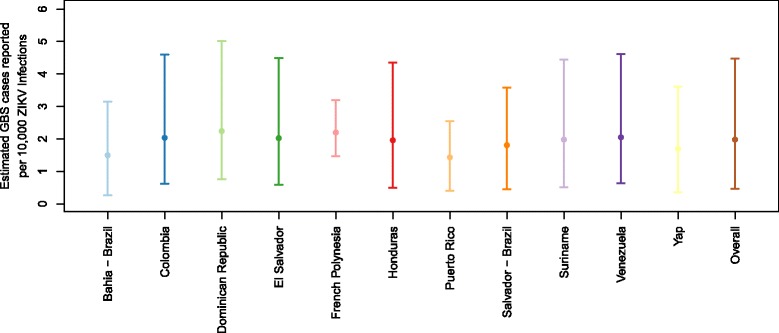
Mean and 95% CrI for the estimated risk of reported GBS related to ZIKV infection at each location and overall

Using reported suspect GBS and Zika case data from all 11 locations and the ZIKV infection incidence estimates for Yap and French Polynesia, we estimated overall GBS risk associated with ZIKV infection, the probability of ZIKV infection *p*_*Z*_, and the probability of a suspect case being reported per ZIKV infection, *p*_*S*_, for each location during the time periods considered. Incorporating data from these 11 locations and accounting for baseline risk, we estimated the location-specific risk (Fig. 2). The across-location minimum and maximum estimates were used to estimate an average risk of becoming a reported GBS case after ZIKV infection across-location of approximately 2.0 GBS cases per 10,000 infections (95% credible interval 0.5–4.5 per 10,000 ZIKV infections). The posterior estimate for the baseline risk of GBS had a median of 0.8 (95% CrI 0.6–1.1) per 100,000 persons per year, slightly lower than the prior median risk of 1.1 (95% CI 0.8–1.9) cases per 100,000 persons per year [4].

To assess the sensitivity of our findings to data from each location, we repeated our analysis by sequentially omitting data from each location and re-estimating the GBS risk as well as the other model parameters. Even though the probability of infection, the risk of GBS due to ZIKV infection, and the number of suspect Zika cases per ZIKV infection were estimated independently for each location, locations were coupled together since the latter two estimated quantities were constrained between bounds that were the same for all places. Results varied little except when data from Yap or French Polynesia were omitted. French Polynesia was unique as the data included both an infection risk estimate and reported GBS cases. In contrast, Yap included an infection risk estimate but no reported GBS cases. Without data from these two locations, the estimated ZIKV infection risk in other locations tended to be much more uncertain (Additional file 1: Figure S1), leading to both lower and higher estimates of GBS risk (Additional file 1: Figure S2).

Our analytical framework related incidence of ZIKV infection to the incidence of reported suspect GBS incidence and reported suspect Zika cases such that we simultaneously estimated location- and time-specific ZIKV infection incidence and the frequency of reported suspect Zika cases per ZIKV infection. While the incidence of ZIKV infection in Yap and French Polynesia was informed by location-specific seroprevalence studies [10, 12, 19, 20], the other locations had no comparable prior information. For the locations without informative priors for the probability of infection during the study period, the estimated mean probability of ZIKV infection during the time periods considered here ranged from 2% to 17% (Table 2). The posterior estimate for the proportion of ZIKV infections that are ultimately reported as suspect cases ranged from 1.0% to 18%. Our sensitivity analysis suggested that the location-specific estimates of infection risk and reported suspect cases per ZIKV infection were stable (Additional file 1: Figures S1 and S3) and were most sensitive to the removal of data either from French Polynesia (as with the estimate for the GBS risk after a ZIKV infection) or from Yap.

**Table 2:** Estimated probability of Zika virus (ZIKV) infection incidence, suspect Zika cases reported per ZIKV, and suspect Guillain–Barré syndrome (GBS) cases per 10,000 ZIKV infections by location

Location	Estimated probability of ZIKV infection, *p*_*Z*_ Mean (95% CrI)	Estimated suspect Zika cases reported per ZIKV infection, *p*_*S*_ Mean (95% CrI)	Estimated GBS cases reported per 10,000 ZIKV infection, *p*_*GZ*_ Mean (95% CrI)
Bahia, Brazil	0.02 (0.01–0.05)	0.14 (0.04–0.25)	1.5 (0.3–3.1)
Colombia	0.09 (0.03–0.23)	0.03 (0.01–0.07)	2.0 (0.6–4.6)
Dominican Republic	0.11 (0.04–0.25)	0.01 (0.00–0.01)	2.2 (0.8–5.0)
El Salvador	0.15 (0.05–0.41)	0.01 (0.00–0.03)	2.0 (0.6–4.5)
French Polynesia	0.64 (0.48–0.78)	0.18 (0.14–0.23)	2.2 (1.5–3.2)
Honduras	0.04 (0.01–0.12)	0.07 (0.02–0.15)	2.0 (0.5–4.3)
Puerto Rico	0.17 (0.08–0.46)	0.15 (0.04–0.26)	1.4 (0.4–2.5)
Salvador, Brazil	0.08 (0.03–0.23)	0.11 (0.03–0.21)	1.8 (0.4–3.6)
Suriname	0.15 (0.04–0.47)	0.06 (0.01–0.15)	2.0 (0.5–4.4)
Venezuela	0.12 (0.04–0.30)	0.01 (0.00–0.03)	2.0 (0.6–4.6)
Yap	0.69 (0.57–0.80)	0.04 (0.03–0.04)	1.7 (0.4–3.6)

*CrI* credible interval

**Fig. 3:**
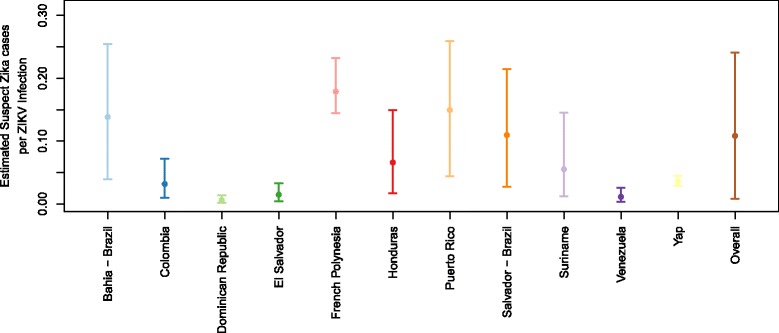
Mean and 95% CrI for the estimated suspect Zika cases reported per ZIKV infection during an outbreak at each location and overall

We estimated the overall expected frequency of reported suspect Zika cases per ZIKV infection based on the bounds estimated across locations, which indicates the expected frequency at unspecified locations to be 0.11 (95% CrI 0.01–0.24) (Fig. 3). By comparing this frequency to the risk of reported GBS, we estimated that, on average, across locations, there are approximately 111 (95% CrI 0–567) reported GBS cases per 10,000 reported suspect Zika cases; this estimate and its credible interval are shown with the grey lines in Fig. 1 and by location in the supplement (Additional file 1: Figure S5).

## Discussion

As of March 2017, GBS had been observed to be temporally associated with Zika outbreaks in 23 countries [1]. We aimed to estimate the risk of GBS related to ZIKV infection given limited ecological-scale data. Although GBS is severe, generally recognizable, and recommendations have been made to standardize case definitions and diagnostic criteria [21], reporting likely varies across countries. To make estimates based on data from 11 different locations, we therefore assumed that, while true ZIKV-associated GBS risk was likely similar across locations, location-specific variability in risk and reporting would lead to variations in the observed number of cases, especially in relation to the observed number of suspect cases.

We evaluated all of our estimates by sequentially removing data from individual locations and obtaining new model estimates. This evaluation revealed that our estimates were most sensitive to the data from French Polynesia. This did not come as a surprise since French Polynesia is the only location with both an estimate of infection incidence and a non-zero number of GBS cases. The Yap outbreak also had an estimate of infection incidence but without any accompanying GBS cases detected. The data from Yap was therefore helpful in estimating the upper bound of reported GBS risk following ZIKV infection, but not in further refining that risk.

Our overall estimate for the risk of reported GBS given ZIKV infection was 2.0 (95% CrI 0.5–4.5) GBS reported cases per 10,000 ZIKV infections, close to the point estimate of 2.4 GBS cases per 10,000 ZIKV infections estimated using only data from French Polynesia [9]. The estimate for French Polynesia was obtained by dividing the 42 reported GBS cases by the estimated total number of infections using an incidence of infection of 66%, as estimated in a serosurvey, and the population size of French Polynesia [9]. When we removed the French Polynesia data, our estimate was somewhat lower, underscoring the importance of the infection prevalence estimates and of considering risk across various locations. In addition, since our model does not account for the correlation between GBS risk and a number of factors such as age and sex [4], we expect discrepancies between our risk estimate and what is observed in realistic public health settings. As data with more detail and from more locations become available, these estimates can be further refined, identifying specific risk groups (e.g., older men [4]) and characterizing the relationship between time of ZIKV infection and GBS onset. Beyond Zika, the current estimates of risk are also of a similar magnitude to the estimated risk of GBS caused by *Campylobacter jejuni* infection – an infection that is well known to be associated with GBS – which is estimated to be between 2.5 and 6.5 GBS cases per 10,000 infections [22].

For each location analyzed, the estimated number of ZIKV-associated reported GBS cases was several times higher than the expected number of reported baseline GBS cases, though the magnitude of the difference varied substantially based on the estimated ZIKV infection incidence. With a high incidence of infection, as in French Polynesia and Yap, the estimated risk of GBS was 25 to 43 times higher during the outbreaks than under typical baseline conditions. For each other location, estimates of incidence over the respective study period were lower (2 to 17%, on average) and indicated significant uncertainty and variability across locations (Table 2 and Additional file 1: Figure S1). These lower estimated incidences may indicate differing epidemiology, geographical heterogeneity within the location, different population densities, varied reporting, and the fact that the data for these locations only represents part of the time period over which the outbreaks occurred. In these locations, Zika-associated reported GBS risk was 1–6 times higher than baseline risk over the time periods of the collected data.

Our mean estimates for the relationship between reported suspect Zika cases and infection incidence ranged from 0.01 to 0.18 suspect cases per ZIKV infection. Differences across locations reflect the relative numbers of GBS and suspect Zika cases reported. For example, the Dominican Republic, Venezuela, and El Salvador all had relatively high numbers of reported GBS cases per suspect Zika case. These differences may be explained by relatively low reporting of suspect Zika cases, high reporting of suspect GBS cases, or a combination of the two. Variation may reflect differences in care-seeking behavior, the availability of public health services, and reporting practices for both Zika and GBS. Across locations, we estimated that 0.11 (95% CrI 0.01–0.24) suspect cases were reported per ZIKV infection, encompassing point estimates from the previous outbreaks. For the outbreak in Yap, only 185 individuals sought care with Zika symptoms even though there were an estimated 5005 individuals infected, such that the number of reported suspect cases represented only approximately 3.7% of the total number of infections [13]. In French Polynesia, over 31,000 suspect cases were reported [23], with an estimated 185,000 infections (based on 66% seroprevalence and a population of approximately 280,000 people [19]). In this case, reported suspect cases represent approximately 17% of the total number of infections. Others have estimated a 94% incidence of infection in that outbreak, in which case suspect cases would be approximately 11.5% of the number of infections [19].

This analysis has a number of limitations, some of which have been noted above. The first is the great variability that exists in reporting practices of Zika and GBS cases across locations and likely over time as the outbreaks unfolded. Furthermore, misclassification of ZIKV infections, or of other illnesses as Zika, certainly occurred in many countries in the Americas with ZIKV outbreaks. An additional limitation was the lack of weekly Zika and GBS case data for most locations. This type of data would have provided many more data points with which to characterize the relationship between ZIKV infections and GBS risk. The limited data also did not allow us to assess the association between GBS risk and other variables such as age and sex. Finally, as noted above, the only outbreaks with data on infection incidence were Yap and French Polynesia and these were also the only two locations with data over the full outbreak. For each other location, estimates of incidence over the respective study period indicated significant uncertainty and variability across locations (Table 2 and Additional file 1: Figure S1).

Without serological data to confirm infection prevalence, it is difficult to identify the reasons for difference in reporting or risk across locations. For example, the relationship between reported Zika and GBS cases in Bahia is clear, but both may have been substantially underreported given the early initiation of that outbreak, leading to a low estimate of infection prevalence. Yet, the number of cases that may have been missed cannot be directly assessed without better estimates of overall infection prevalence. If population-level serological data become available, the data could be directly incorporated in the model, as was done for Yap and French Polynesia, allowing the estimates to be informed by these additional data and providing a further pathway to evaluate the estimates.

## Conclusions

With very few well-described historical Zika outbreaks, much uncertainty remains regarding the association between ZIKV infection, clinical illness, and GBS. Our analysis provides a framework that leverages data from diverse locations to generate preliminary estimates that account for wide variability and uncertainty. These estimates put general bounds on the relationship between reported suspect Zika cases, reported GBS cases, and the overall incidence of ZIKV infection. The findings support the observation that, during a ZIKV outbreak, GBS incidence could be many times higher than normal, and that risk may be similar across locations despite discrepancies in the number of GBS cases reported per suspect Zika case. As the current Zika epidemic progresses, more reports of GBS cases are likely to occur; these risk estimates can help inform public health preparedness activities to ensure that adequate resources are available when those cases occur.

## Additional file

Additional file 1: Reported suspect Zika and Guillain–Barré syndrome (GBS) case data. Bayesian inference model of clinical GBS cases arising from Zika infections. Description of data: Additional file gives technical details of our Bayesian inference framework for the estimation of GBS risk following a ZIKV infection. (PDF 2723 kb)

## Abbreviations

GBS: Guillain–Barré syndrome
ZIKV: Zika virus
