# Supplementary material for: Guillain–Barré syndrome risk among individuals infected with Zika virus: a multi-country assessment
Source: BMC Med. 2018 May 15;16:67. doi: 10.1186/s12916-018-1052-4 (PMC5952697; doi:10.1186/s12916-018-1052-4)
Supplement: Supplementary file 1 — Reported suspect Zika and Guillain–Barré syndrome (GBS) case data. Bayesian inference model of clinical GBS cases arising from Zika infections. Description of data: Additional file gives technical details of our Bayesian inference framework for the estimation of GBS risk following a ZIKV infection. (PDF 2723 kb) [file 12916_2018_1052_MOESM1_ESM.pdf]

Additional File 1

Reported Suspect Zika and Guillain-Barré Syndrome Case Data

We gathered case data from eleven locations with confirmed Zika outbreaks and reports of potentially associated Guillain Barré Syndomre (GBS) cases with the goal of estimating the risk of GBS and the number of suspect reported Zika cases per Zika virus (ZIKV) infection (Table 1 in the main text). The data obtained represent total case counts over time spans that vary by location. The data are publicly available and was obtained from either weekly epidemiological bulletins or published research articles: Yap [1], French Polynesia [2], Bahia state in Brazil [3], Colombia [4], Dominican Republic [5], El Salvador [3], Honduras [3], Puerto Rico [6] and Salvador city in Brazil [7], Suriname [3] and Venezuela [3]. It should be noted that all GBS cases reported in Colombia and Puerto Rico displayed symptoms compatible with ZIKV infections. Despite limited specificity and varying surveillance systems [8], suspect cases were used for consistency and variation in surveillance was considered in the model framework.

Weekly reports from Puerto Rico aggregate all arboviral disease suspect cases together, but classify confirmed cases as dengue, chikungunya or Zika. To estimate the number of Zika cases, we assumed that among suspect cases, Zika represents the same proportion of the total as in confirmed cases.

Bayesian Inference Model of Clinical Guillain-Barré Syndrome Cases arising from Zika Virus Infections

Here, we describe in detail the mathematical formulation of our Bayesian inference model of Guillain-Barré Syndrome cases arising from Zika virus infections. The model employed Markov Chain Monte Carlo (MCMC) sampling and was implemented in JAGS through the package 'rjags' in R.

We consider that at each location ℓ with population N_ℓ the total number of Zika infections Z_ℓ during a certain time period, as well as the ones that give rise to reported suspect cases S_ℓ are Binomially distributed as:

$$Z_\ell \sim \text{Bin}(p_{Z\,\ell}, N_\ell), \tag{1a}$$

$$S_\ell \sim \text{Bin}(p_{S\,\ell}, Z_\ell), \tag{1b}$$

where, at location ℓ, the unknown probabilities $p_{Z\,\ell}$ and $p_{S\,\ell}$ denote the probability of infection and the proportion of overall ZIKV infections that result in reported suspect cases, respectively.

Seroprevalence studies in French Polynesia resulted in a 95% confidence interval (CI) for the estimated ZIKV infection of 0.42–0.57 in the general population and of 0.60 – 0.71 in schoolchildren [9]. We combine these two estimates and assume that

the 95% CI of the population fraction infected with ZIKV in French Polynesia was between 0.42 – 0.71. In Yap, analogous seroprevalence studies resulted in the range 0.68–0.77 for the 95% CI for the infected fraction of the population [1]. However, no similar studies informing the infection probabilities at the other locations exist. We select the prior distributions for $p_{Z\,\ell}$ to be

$$p_{Z\,\ell} \sim \mathrm{Beta}(a_{Z\,\ell}, b_{Z\,\ell}). \tag{2}$$

Where $a_{Z\,\ell}$ and $b_{Z\,\ell}$ are selected via the method of moments, choosing location-dependent means and variances, so as to match those of a uniform random variable with the possible range of attack rates (0.42–0.71 and 0.68–0.77 for French Polynesia and Yap, respectively, and 0.0–1.0 for all other locations). For French Polynesia and Yap, this process results in prior distributions informed by the ranges observed in the seroprevalence studies but that nevertheless allow for values outside those ranges.

We assume that the risks of developing GBS ($p_{GZ\,\ell}$) and symptoms ($p_{S\,\ell}$) after an infection with ZIKV are similar across locations but that reporting rates may vary more drastically. The location-specific differences in p_{GZ} and p_S are accounted for by giving $p_{GZ\,\ell}$ and $p_{S\,\ell}$ hyperpriors reflecting location-independent bounds estimated by our framework:

$$\begin{aligned} p_{GZ\,\ell} &\sim \mathrm{Unif}(p_{GZ\,\min}, p_{GZ\,\max}), & p_{S\,\ell} &\sim \mathrm{Unif}(p_{S\,\min}, p_{S\,\max}),\\ p_{GZ\,\min} &\sim \mathrm{Unif}(0,1), & p_{S\,\min} &\sim \mathrm{Unif}(0,1),\\ p_{GZ\,\max} &\sim \mathrm{Unif}(p_{GZ\,\min}, 1), & p_{S\,\max} &\sim \mathrm{Unif}(p_{S\,\min}, 1). \end{aligned}$$

In addition, the location-independent bounds $p_{S\min}$, $p_{S\max}$, $p_{GZ\min}$ and $p_{GZ\max}$ are used to generate overall, average estimates of the risk of being reported as a Zika and GBS case, respectively.

For Dominican Republic, French Polynesia, Salvador–Brazil and Yap, we consider GBS cases arise from either: (i) infections with Zika virus or (ii) from all other reasons (termed the GBS 'baseline'), or from both simultaneously. For Colombia and Puerto Rico we disregard the baseline origin for GBS cases since the data for those two places only includes individuals with a previous illness compatible with Zika.

The observed baseline GBS rate has a median of 1.1 cases per 100,000 population per year and ranges from and 0.8–1.9 cases per 100,000 per year [10]. We interpret this as a median number of GBS cases of 2.1 (95% CI: 1.6–3.7) per 10 million population on a weekly basis. This weekly baseline rate is assigned a Beta prior that was fit to these statsitical properties:

$$p_{\text{GBS Baseline}} \sim \mathrm{Beta}(12.5,\ 5.7 \times 10^7). \tag{4}$$

Putting all pieces together, our model estimates, at each location, the aggregate number of GBS cases from all possible causes that arise binomially from the whole

population of N_ℓ individuals:

$$G_\ell \sim \mathrm{Bin}\Big((1 - p_{\text{GBS Baseline}} D_\ell) \cdot p_{GZ\ell} \cdot p_{Z\ell} + p_{\text{GBS Baseline}} D_\ell, N_\ell\Big), \tag{5}$$

where G_ℓ is the total number of reported GBS cases over the D_ℓ weeks of the outbreak at the given location ℓ. Note that $p_{\text{GBS Baseline}}$ is multiplied by D_ℓ to calculate a probability of baseline GBS for the time period corresponding to the data for each location.

Supplementary Figures

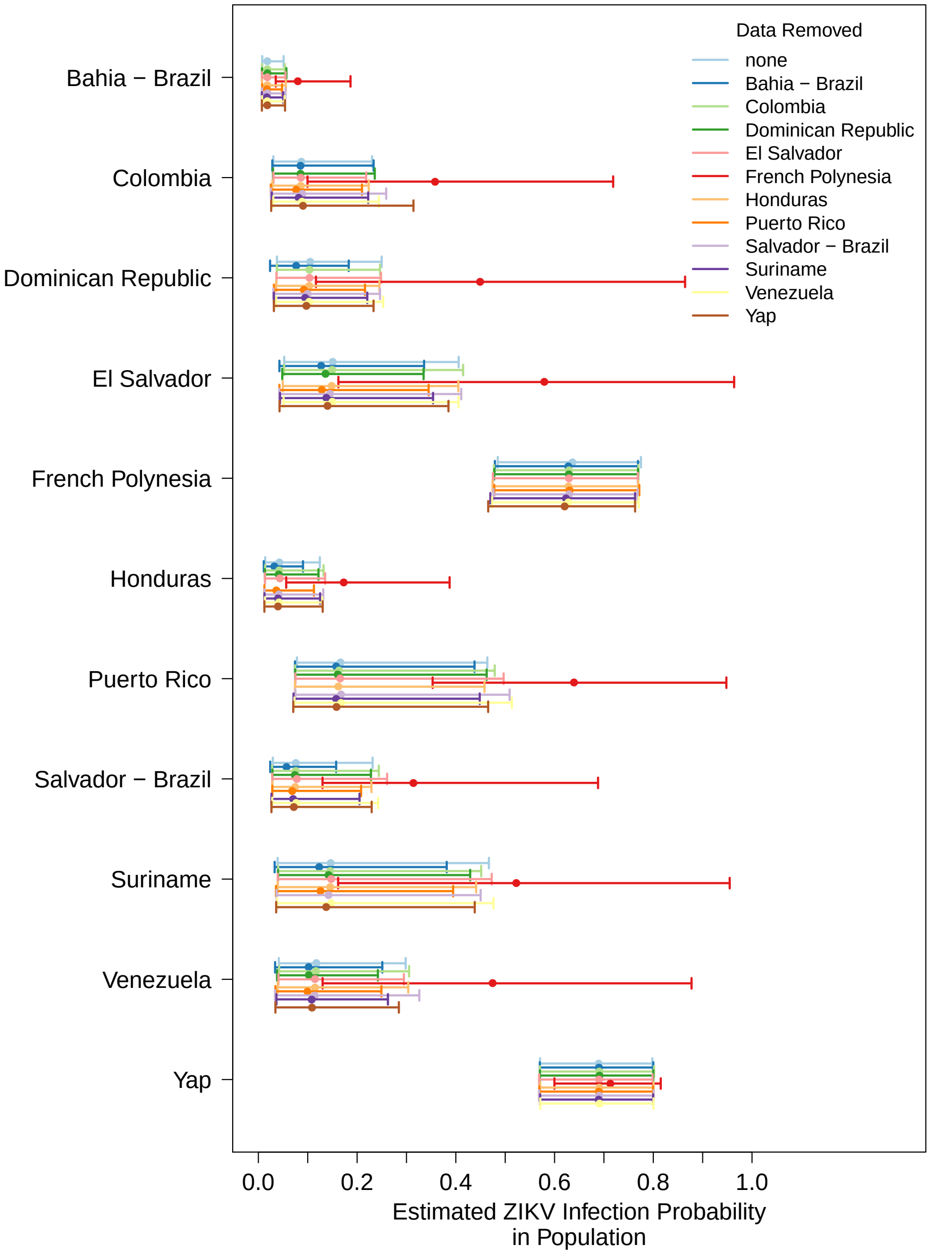

Figure S1: ZIKV infection probabilities estimated for different locations, with different data elements omitted. Points denote means of our simulation runs, error bars indicate 95% credible intervals.

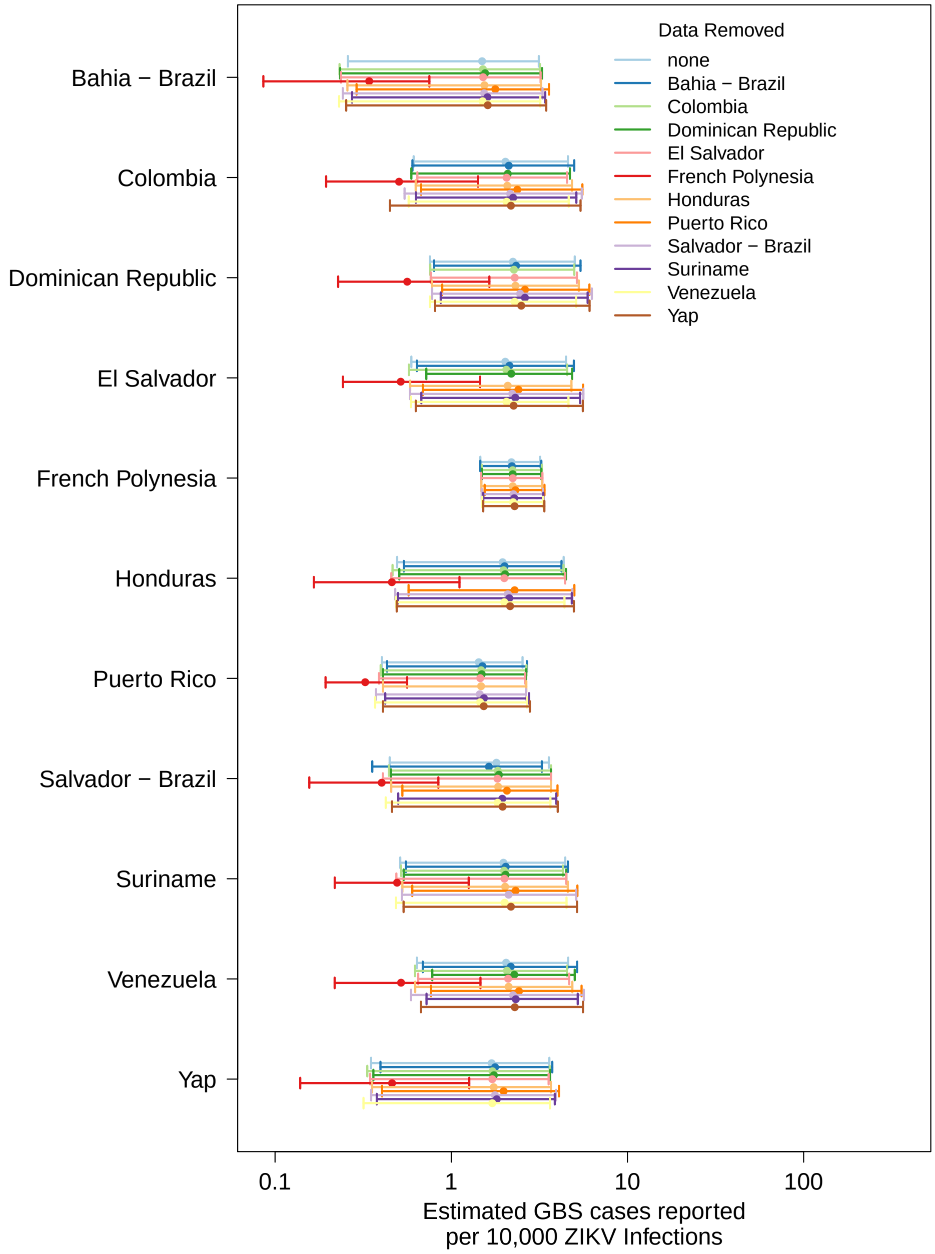

Figure S2: Risk of GBS per 10,000 ZIKV infections, as estimated with different data omitted. Points denote means of our simulation runs, error bars indicate 95% credible intervals.

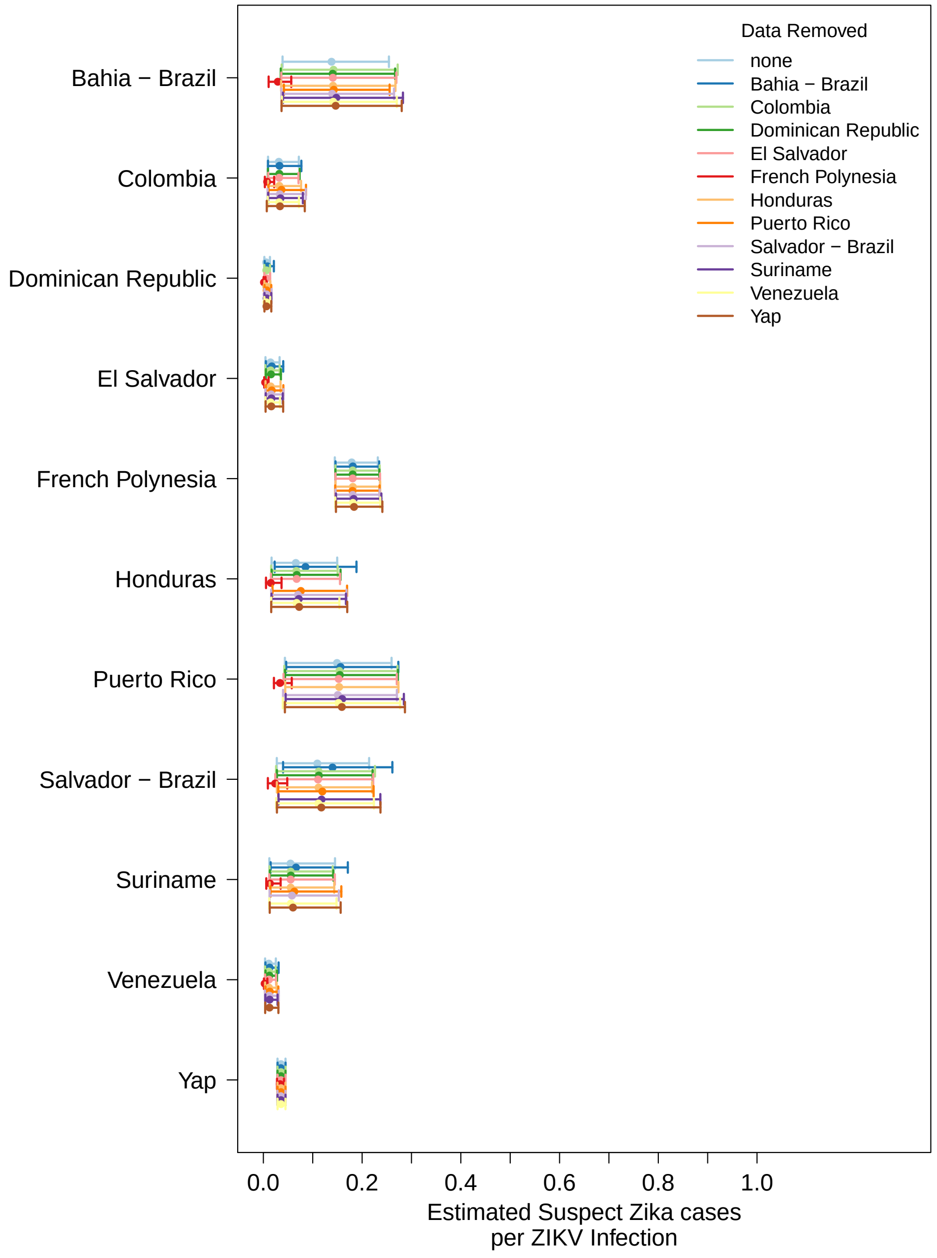

Figure S3: Estimated suspect Zika cases reported per ZIKV infection for different locations, with different data elements omitted. Points denote means of our simulation runs, error bars indicate 95% credible intervals.

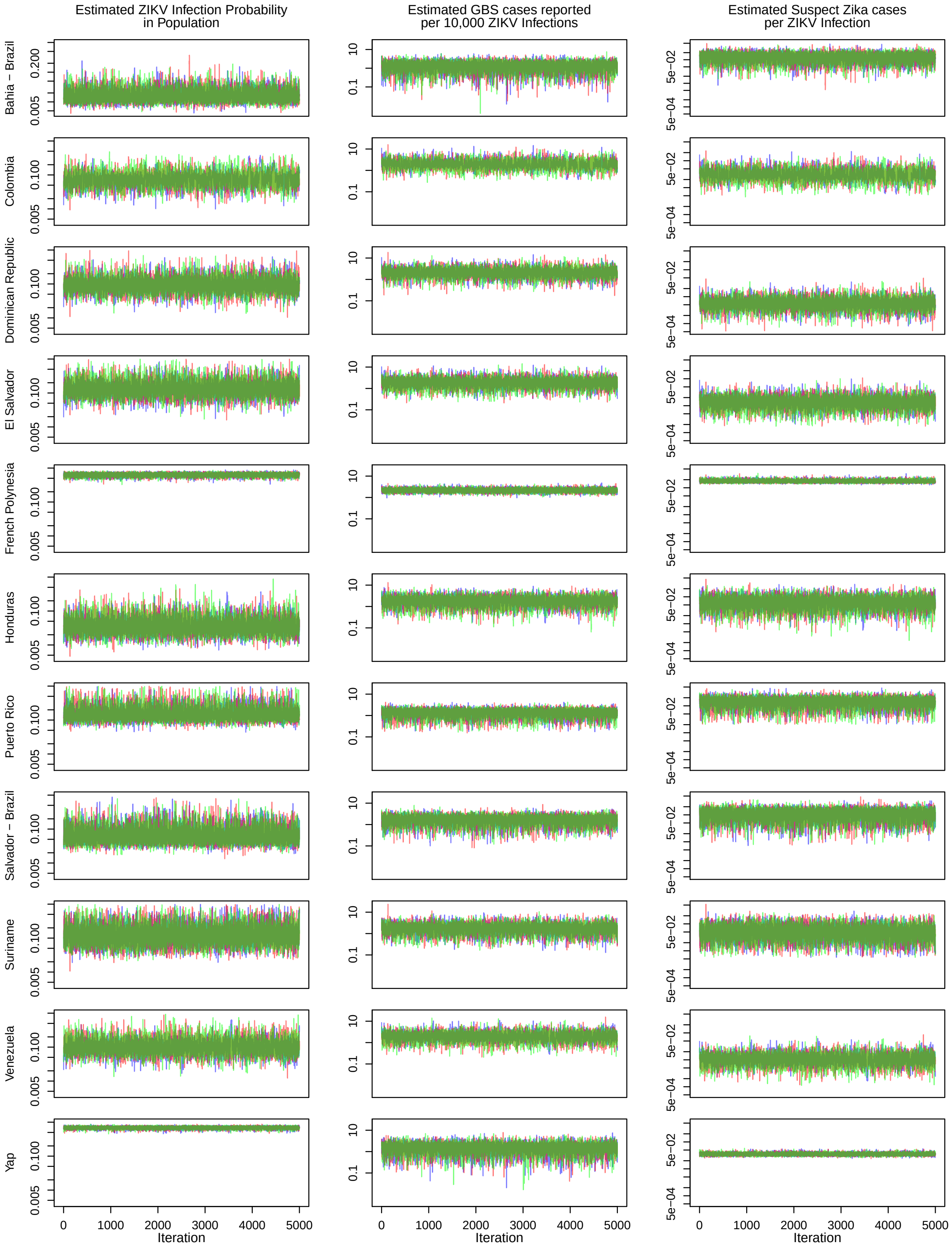

Figure S4: Convergence graphs for the three variables p_Z, p_{GZ} and p_S in the left, center and right columns, respectively, across different locations (rows). Three chains are displayed in blue, red and green.

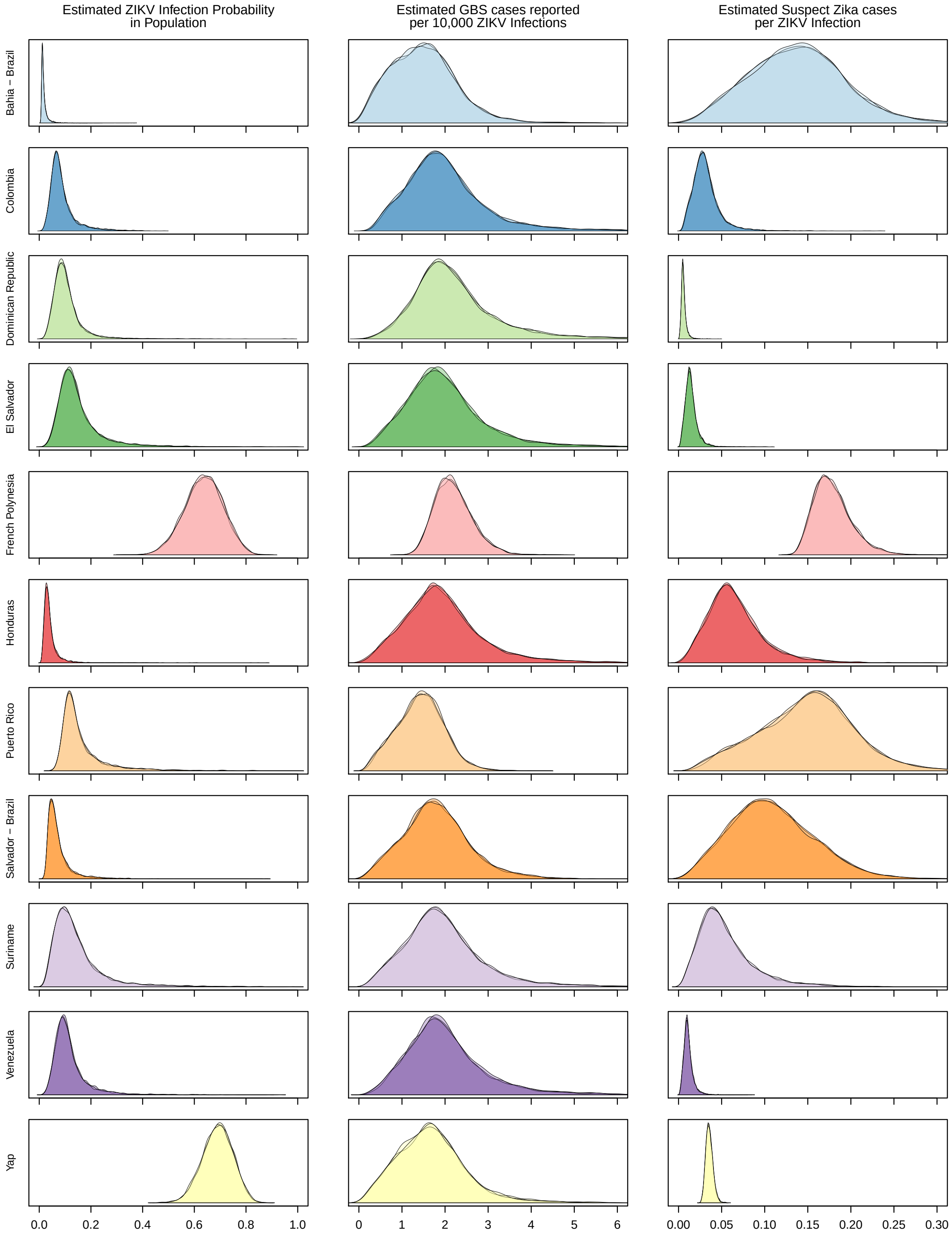

Figure S5: Densities for the three independent chain simulations of the variables p_Z, p_{GZ} and p_S in the left, center and right columns, respectively, across different locations (rows).

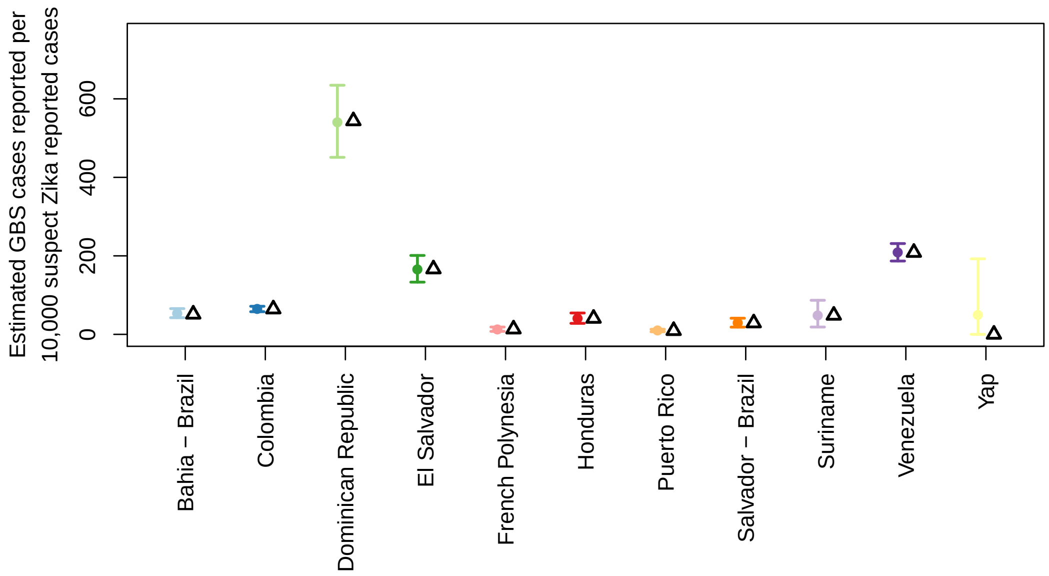

Figure S6: Estimated GBS risk per 10,000 suspect ZIKV cases reported. Points denote means of our simulation runs, error bars indicate 95% credible intervals; black triangles denote epidemiological data.

References

[1] Duffy, M.R., et al, "Zika virus outbreak on Yap Island, Federated States of Micronesia," *N Engl J Med*, vol. 360(24), pp. 2536–43, 2009.

[2] Cao-Lormeau, V.M., et al., "Guillain-Barre Syndrome outbreak associated with Zika virus infection in French Polynesia: a case-control study," *Lancet*, 387(10027): p. 1531-9, 2016.

[3] Dos Santos, T., et al., "Zika Virus and the Guillain-Barre Syndrome - Case Series from Seven Countries", *N Engl J Med*,375(16): p. 1598-1601, 2016.

[4] "Boletin Epidemiologico Semanal", semana 52, *Instituto Nacional de Salud, Colombia*, 2016.

[5] "Boletin Epidemiologico Semanal", semana 52, *Ministerio de Salud Publica, Republica Dominicana*, 2016.

[6] "Informe Semanal de Enfermedades Arbovirales", semana 7, *Departamento de Salud de Puerto Rico*, 2017.

[7] Paploski, I.A., et al., "Time Lags between Exanthematous Illness Attributed to Zika Virus, Guillain-Barre Syndrome, and Microcephaly, Salvador, Brazil", *Emerg Infect Dis*, 22(8): p. 1438-44, 2016.

[8] Ogden, N.H., et al. "Risk of travel-related cases of Zika virus infection is predicted by transmission intensity in outbreak-affected countries", *Parasites & Vectors*, 10:41, 2017.

[9] Aubry, M., et al. "Zika Virus Seroprevalence, French Polynesia, 20142015," *Emerg Infect Dis.*, vol. 23(4), pp:669–672, 2017.

[10] Sejvar, J.J. and Baughman A.L. and Wise M. and Morgan O.W., "Population Incidence of Guillain-Barré Syndrome: A Systematic Review and Meta-Analysis," *Neuroepidemiology*, vol. 36, pp. 123–133, 2011.
